# A Fixed-Threshold Approach to Generate High-Resolution Vegetation Maps for IKONOS Imagery

**DOI:** 10.3390/s8074308

**Published:** 2008-07-25

**Authors:** Wen-Chun Cheng, Jyh-Chian Chang, Chien-Ping Chang, Yu Su, Te-Ming Tu

**Affiliations:** 1 Department of Electrical Engineering, Chung Cheng Institute of Technology, National Defense University, Taoyuan 335, Taiwan, R.O.C.; E-mails: alex.cherng@gmail.com; cpchang@ccit.edu.tw; suyu@ccit.edu.tw; tutm@ccit.edu.tw; 2 Department of Information Communications, Kainan University, Taoyuan 338, Taiwan, R.O.C.; E-mail: garrychang@mail.knu.edu.tw

**Keywords:** IKONOS, NDVI, Tasseled Cap transformation, image fusion

## Abstract

Vegetation distribution maps from remote sensors play an important role in urban planning, environmental protecting and related policy making. The normalized difference vegetation index (NDVI) is the most popular approach to generate vegetation maps for remote sensing imagery. However, NDVI is usually used to generate lower resolution vegetation maps, and particularly the threshold needs to be chosen manually for extracting required vegetation information. To tackle this threshold selection problem for IKONOS imagery, a fixed-threshold approach is developed in this work, which integrates with an extended Tasseled Cap transformation and a designed image fusion method to generate high-resolution (1-meter) vegetation maps. Our experimental results are promising and show it can generate more accurate and useful vegetation maps for IKONOS imagery.

## 1. Introduction

In the past decades, a considerable number of new technologies and methods to generate vegetation maps from remote sensing imagery had been developed, including a variety of sensors cooperating with different scale imagery that is interesting and important to urban planners and land managers [1]. To generate a vegetation index (VI) using the spectrum characteristics of sensors, Jordan [2] used the ratio of near infrared to red to estimate leaf biomass. After that, the normalized difference vegetation index (NDVI) was used for Landsat MSS (Multispectral Scanner) data by Rouse [3] and this became the primary means to evaluate green vegetation properties. Nowadays, there are more and more remote sensing systems with various sensors deployed and varying in spatial resolution, radiometric precision, temporal coverage and spectral characteristics. Therefore, to increase the accuracy and consistency of land cover measurements for each sensor system, the differences among various types of sensors must be considered [4]. In other words, the NDVI needs to be calibrated for different sensor instruments [5], even though it has accomplished promising performance in some applications of vegetation classification. In addition, the NDVI requires a threshold to distinguish vegetated areas from other surface types, e.g. the NDVI of an area containing dense vegetation will tend to positive values (say 0.3 to 0.8) while soils will be characterized by rather small positive values (say 0.1 to 0.2). Furthermore, the calculation of the NDVI value turns out to be sensitive to a number of factors which are difficult to be controlled and estimated simultaneously when the images are collected in various conditions, such as clouds, atmosphere, soil conditions, and so on. Thus, the threshold selection is difficult for extracting vegetation information from diverse scenes.

Moreover, after IKONOS was launched and started to provide high-resolution imagery (4-meter multispectral and 1-meter panchromatic), most details of buildings, individual trees, and vegetation structural variations can be well detected with 1-meter spatial resolution images. It provides a new data source for monitoring agricultural production, and giving information for the development of crops during the growing season [7]. With the objectives of vegetation visualization, Malpica [8] developed a new fusion approach which consists of a hue spectral adjustment by using the NDVI of imagery for vegetation enhancement. In the fused image, the deciduous and evergreen vegetation can be clearly differentiated. Furthermore, to allow easier identification of distinct ground surface types, Horne [9] had examined the underlying structure on a pixel-by-pixel basis, and presented an IKONOS Tasseled Cap transformation (TCT) approach. By taking 195 IKONOS images from various sets of environment, the IKONOS TCT can quickly cluster most vegetation information into a single component. However, even though the TCT has been calibrated for IKONOS imagery, it also has the same threshold selection problem as NDVI. That is, a proper threshold is not easy to be decided for extracting vegetation information.

To cope with this problem, we propose a new method to generate a high-resolution and better visual-interpretation vegetation map for IKONOS imagery with a fixed threshold. The presented approach extends the original IKONOS TCT and integrates with a fast fusion technique to increase the spatial details of the vegetation map. The experimental results demonstrate that the proposed approach can generate a high-resolution and more discriminable vegetation map for IKONOS imagery efficiently.

## 2. Fixed-Threshold Approach of Vegetation Map Generation

The well-known NDVI method is given by:

(1) $$\text{NDVI}=\frac{\left(\text{NIR}-R\right)}{\left(\text{NIR}+R\right)}$$

where *R* and *NIR* are the reflectance for red and near infrared spectral bands, respectively. Because NDVI was originally developed for the Landsat MSS data (an example of coarse resolution imagery), it is rarely used to generate high-resolution (1-meter) vegetation maps directly. Meanwhile, selecting an appropriate threshold value is also difficult in various scenarios for NDVI. Some examples of the histograms of NDVI and their corresponding thresholds are shown in Section 3.

An alternative approach for vegetation extraction is the TCT, which was derived in 1976 [10] for the four bands of the Landsat MSS sensor. The analysis was extended to the Landsat TM (Thematic Mapper) sensor in 1984 [11]. For IKONOS imagery, Horne developed the IKONOS Tasseled Cap coefficients by analyzing of a set of 195 IKONOS images [9], they are given by:

(2) $$\begin{array}{c} TC_{1}=0.326\;B+0.509\;G+0.560\;R+0.576\;\text{NIR}\\ TC_{2}=-0.311\;B-0.356\;G-0.325\;R+0.819\;\text{NIR}\\ TC_{3}=-0.612\;B-0.312\;G+0.722\;R-0.081\;\text{NIR}\\ TC_{4}=-0.650\;B+0.719\;G-0.243\;R-0.031\;\text{NIR} \end{array}$$

where *R*, *G*, *B* and *NIR* are the red, green, blue and near infrared bands, respectively.

The first component of TC (*TC*_1_) can be considered the sum of the original bands, and it is very similar to the panchromatic (Pan) image. The second component (*TC*_2_) can be treated as the near infrared band minus the visible bands. It becomes major information to distinguish different surface types, while the vegetation data tend to be strong, and the roads and buildings tend to be weak. In other words, all man-made objects are presented close to dark while all vegetation, e.g. grass and trees, is near bright. The third component (*TC*_3_) can be regarded as red minus blue, while the fourth component (*TC*_4_) as green minus blue. The last two components should be useful for further distinguishing between vegetation and soil. However, the fourth component is low in variance, about 0.2% of the total variance, usually as small as noise, and it can be ignored in most cases [9]. Generally, a TCT image is composed of component *TC*_1_ (R), *TC*_2_ (G) and *TC*_3_ (B).

As presented, the second component contains most vegetation information while the first and third components contain other types of survey information. To verify the robustness of IKONOS TCT approach, 126 images were tested and all of them complied with the characteristic of each component as presented in (2). Motivated from this, an enhanced vegetation index, *VI_TC_*, can be derived from emphasizing the second component and depressing the other two components. That is:

(3) $$VI_{TC}=a\cdot TC_{2}-b\cdot TC_{1}-c\cdot TC_{3}\;,$$

where *a*, *b* and *c* are the weighting factors. Generally, the data dependent regression approach is used to obtain the weighting factor *a*, *b* and *c*, but it is computationally intensive for huge remote sensing imagery. To reduce the computational complexity, we apply a well-known pseudo Karhunen-Loéve transform (PKLT) [12] which is a data independent approach to replace the data dependent method, Principal Component Analysis (PCA). The PKLT is represented by:

(4) $$\left[\begin{array}{c} PC_{1}\\ PC_{2}\\ Pc_{3} \end{array}\right]=\left[\begin{array}{ccc} 1/3 & 1/3 & 1/3\\ 1/2 & 0 & -1/2\\ -1/4 & 1/2 & -1/4 \end{array}\right]\;\left[\begin{array}{c} TC_{1}\\ TC_{2}\\ TC_{3} \end{array}\right]$$

Here, for vegetation extraction, we only utilize the operation of component *PC_3_* which fits our requirement, depressing the first and third components, and emphasizing the second component. That is:

(5) $$PC_{3}=-\frac{1}{4}TC_{1}+\frac{1}{2}\cdot TC_{2}-\frac{1}{4}TC_{3}$$

Therefore, $VI_{TC}=\frac{1}{2}\cdot TC_{2}-\frac{1}{4}TC_{1}-\frac{1}{4}TC_{3}$, and the vegetation map, *V_map_*, can be produced by a semi-threshold approach. That is:

(6) $$V_{\text{map}}=\{\begin{array}{l} VI_{TC},\text{where}\;VI_{TC}\geq \theta \\ 0,\;\text{where}\;VI_{TC}<\theta \end{array}$$

After observing 126 IKONOS images over difference scenes, the threshold *q* is selected and set at a fixed value, 0. Then, the ground survey information can be separated into two parts, the foreground for vegetation information and the background for all non-vegetation information. Some demonstrative examples of the histograms of *VI_TC_* and the corresponding vegetation maps generated by the proposed approach are shown in the following section.

To take the advantage of high spatial resolution information of the Pan images, a high-resolution vegetation map can be generated by fusing with a Pan image and a lower resolution *V_map_*. For doing so, the lower resolution *V_map_* should firstly be resized to an image *V_map_*′ with the same scale as the Pan image by cubic convolution. Hence, a pseudo color image *T* can be composed by

(7) $$T=\left[\begin{array}{c} R^{\prime }\\ G^{\prime }\\ B^{\prime } \end{array}\right]=\left[\begin{array}{c} 0\\ V_{\text{map}}\prime \\ 0 \end{array}\right]$$

where the image *V_map_′* represents the green band of the pseudo color image, and the 0 represents a full black image in red (*R′*) or blue (*B′*) band. Then, the pseudo-color image can be fused with the Pan image to obtain a high-resolution (1-meter) vegetation map which contains all details of the Pan image. To derive the desired fused image, there are many image fusion methods that can be used [13, 14]. Here, a simple and fast version of IHS fusion [15] is used, and it can be implemented by:

(8) $$\left[\begin{array}{c} R_{\text{new}}\\ G_{\text{new}}\\ B_{\text{new}} \end{array}\right]=\left[\begin{array}{c} R^{\prime }\\ G^{\prime }\\ B^{\prime } \end{array}\right]+\left[\begin{array}{c} \delta \\ \delta \\ \delta \end{array}\right],$$

where $\delta =\text{Pan}-\text{I}=\text{Pan}-\frac{V_{\text{map}}\prime }{3}$, $\frac{V_{\text{map}}\prime }{3}$ is the average of *T* =[*R G B′*]*^T^*, the pseudo color image. After all these processes, this final result image can be generated, where only vegetation is shown in green and the other regions are displayed in gray. Some examples will be demonstrated in Section 3. Also, a high-resolution vegetation map of NDVI can be generated by the same fusion procedures.

## 3. Experimental Results

**Figure 1.**
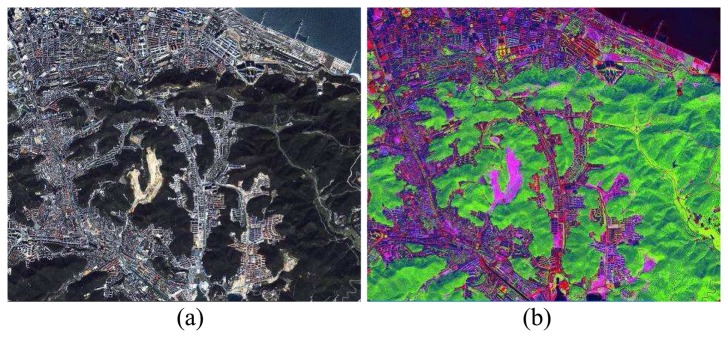
(a) A nature color IKONOS image of Dalian, Liaoning, China. (b) The corresponding IKONOS TCT image of the MS image.

For verification purpose, 126 IKONOS images had been performed with the proposed approach. One of them was chosen as the demonstrative image in Figure 1, which is around the area of Dalian, Liaoning, China, and was taken on Sept. 2003. Figure 1(a) shows the red, green, blue bands of a 4-meter IKONOS MS image. Figure 1(b) shows the IKONOS TCT image of the MS image which had been mostly clustered into few different classes, e.g. buildings, roads, soil, and vegetation, while vegetation area has been marked in green color.

**Figure 2.**
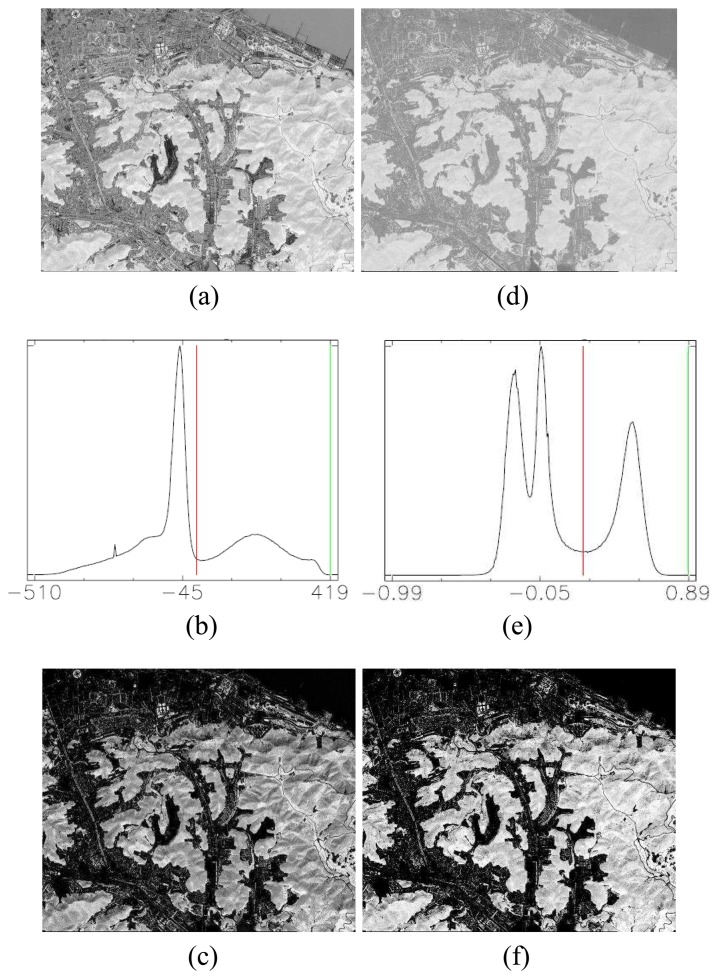
(a) The enhanced vegetation index image in grayscale. (b) The histogram of enhanced vegetation index. (c) The vegetation map generated by proposed semi-threshold approach. (d) The corresponding NDVI image in grayscale. (e) The histogram of NDVI image. (f) The NDVI vegetation map generated with the threshold at 0.22.

**Figure 3.**
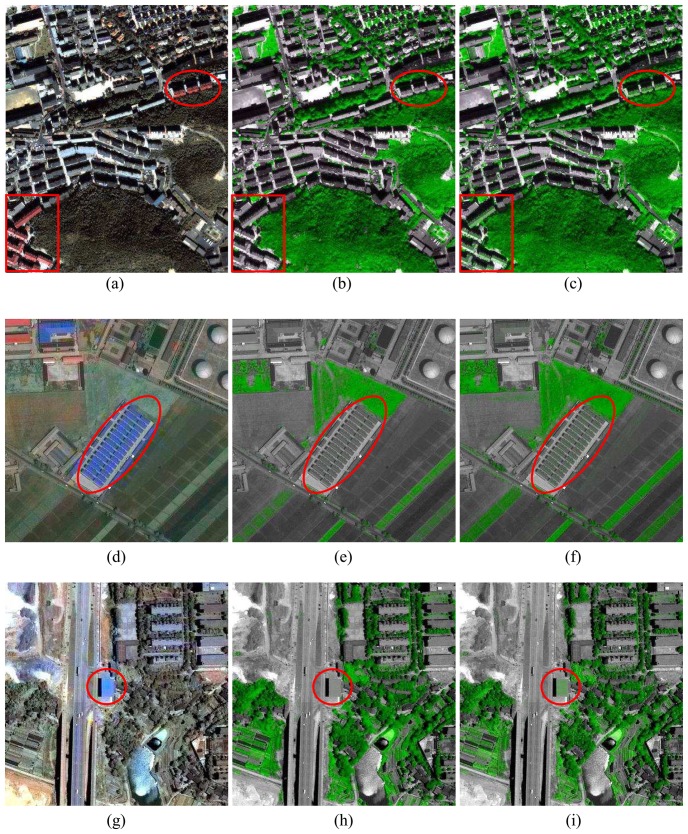
(a), (d) and (g): The chip images of the pan-sharpened image for comparison reference. (b), (e), and (h): The corresponding chips of the high-resolution vegetation map from the proposed approach. (c), (f), and (i): The corresponding chips of the high-resolution vegetation map from NDVI. There are some false alarms within the red squares and circles.

Then, the enhanced vegetation index (*VI_TC_*) was derived from (5) as shown in Figure 2(a) and its histogram is shown in Figure 2(b). Figure 2(c) shows the vegetation map *V_map_* in grayscale image with all vegetation information extracted by (6), while using 0 as the threshold as presented. All preserved vegetation information is shown in light, while others are eliminated and shown in dark. In contrast with NDVI, the image of NDVI and its histogram are shown in Figures 2(d) and (e), respectively. From the observations in our experiments, the thresholds of NDVI are difficult to be manually decided for the 126 tests. However, due to the vegetation area is around two-fifth of whole image and may fall into the first group of the histogram image, a visualized optimal threshold, 0.22, was manually and carefully selected for this scene. The resultant vegetation map is shown in Figure 2(f). Generally, NDVI has similar classified result with our proposed approach in overview. However, there are some false alarms occurred on specific buildings in the NDVI approach. Because of the lack of the ground truth data for the 126 test images, we had visually inspected all test images and found false alarms occurred in 17 images with NDVI approaches. From our experimental observation, some manmade structures which are surrounded by or near a large vegetation area would be likely indicated as vegetation area by NDVI. To illustrate these false alarms more evidently, three sets of small demonstration chips are shown in Figure 3.

Figure 3 shows three sets of small chips over the different areas. The first set of images covers an urban area in the upper portion and a mountain area in the lower part. Figure 3(a) shows the pan-sharpened image with nature color for reference purpose. By (7) and (8), the high-resolution vegetation maps from the proposed approach and the NDVI method are shown in Figures 3(b) and (c), respectively. Both of them can detect well the vegetation area in the urban (grass) or mountain (forest) area. However, NDVI has some false alarms in specific areas which are indicated by the red square and circle, where some roofs with orange color painted in the original image [Figure 3(a)] had been marked with green and misinterpreted as vegetation areas. Furthermore, another two sets of small chips are shown as Figures 3(d) ∼ (i). Obviously, there are some false alarms occurred within the circles in NDVI images as well. That is, those manmade structures with blue painting are marked as vegetation areas.

**Figure 4.**
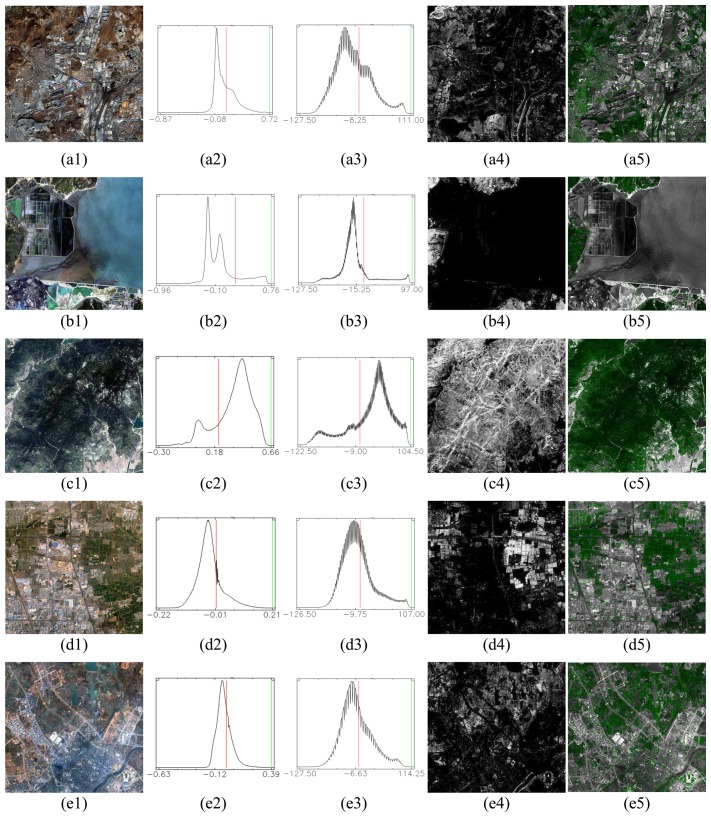
5 IKONOS image sets of different type scenes. (a1∼e1): the pan-sharpened images; (a2∼e2): the histogram of NDVI for each image; (a3∼e3): the histograms of VI_TC_ for each image; (a4∼e4): the vegetation maps generated by the proposed approach; (a5∼e5): the high-resolution vegetation maps fused with the corresponding Pan images.

For further demonstration of the feasibility of the proposed approach, 5 IKONOS image sets from different types of scenes were selected (from the 126 test images) and shown in Figure 4, from (a) to (e) respectively. Figures 4(a1∼e1) show the pan-sharpened images for reference. For comparison purpose, the histograms of the corresponding NDVI images are shown in Figures 4(a2∼e2) and their threshold values are 0.10, 0.19, 0.21, 0.01 and -0.01, respectively. Clearly, t is difficult to decide which is the proper threshold for each image. Figures 4(a3∼e3) are the histograms of the enhanced vegetation index (*VI_TC_*), and the corresponding vegetation maps (*V_map_*) are shown in Figures 4(a4∼e4). All vegetation information can be extracted well at the fixed threshold, 0. The high-resolution vegetation maps which were obtained by fusing *V_map_* with the corresponding Pan images are shown as in Figures 4 (a5∼e5). All vegetation areas had been clearly marked in green, including trees in mountains and grass in urban areas, and all other surfaces are shown in grayscale.

## 4. Conclusions

For vegetation mapping, the NDVI approach has been widely utilized with various sensors. However, the different characteristic of sensors would affect the classification results. Furthermore, the threshold selection is difficult for extracting vegetation information from various scenes. In this work, we have proposed an approach with a fixed-threshold scheme and an image fusion algorithm, which can be suitable for IKONOS imagery, and it is illustrated with more accurate results than NDVI. Moreover, the produced high resolution vegetation maps can provide a better visual understanding for vegetation distribution on ground survey. The fast fusion technique can be used to increase the spatial details for the vegetation maps generated from high-resolution satellite images, such as those from IKONOS. Our experiment results with 126 IKONOS images support the theoretical inference of the proposed approach. In future work, we plan to extend the proposed approach to other high-resolution satellite images, e.g. QuickBird images, and to find a solid theoretical support for the fixed-threshold scheme.
